# Wondering Awe Is the Mediator of the Link Between Experience of Nature and Psychological Wellbeing—Relevance for Public Health

**DOI:** 10.3390/ijerph22111679

**Published:** 2025-11-05

**Authors:** Arndt Büssing, Julia Wilhelm, Daniela Rodrigues Recchia

**Affiliations:** Professorship Quality of Life, Spirituality and Coping, Department of Health, University of Witten/Herdecke, 58313 Herdecke, Germany; julia.wilhelm@uni-wh.de (J.W.); daniela.rodriguesrecchia@uni-wh.de (D.R.R.)

**Keywords:** experience of nature, nature-relatedness, wellbeing, gratitude, wondering awe, availability of green spaces, sporting activities, hiking and trolling

## Abstract

Background: Access to green spaces is crucial for public health. For psychological health, the ability to pause in fascination or wondering awe (as an indicator of mindful resonance with nature) appears particularly relevant. However, it remains unclear whether non-interventional experience of nature is directly related to wellbeing or requires a mediator. Methods: A cross-sectional anonymous survey was conducted among 491 participants (74% women; mean age 51 ± 13 years) with standardized instruments (NR-6, ENS, GrAw-7, GQ-6, WHO-5). Results: Experience of Nature (r = 0.56) and Nature-Relatedness (r = 0.55) are strongly associated with Awe/Gratitude, while only Awe/Gratitude is moderately related to Wellbeing (r = 0.42). Mediator analyses revealed that the relationship between Experience of Nature and Wellbeing is significantly mediated by Awe/Gratitude (β = 2.28, *p* < 0.001). This highlights the central role of this resource through which nature experiences promote wellbeing. Regression analyses confirmed Awe/Gratitude as the best predictor of Wellbeing, followed by Gratitude disposition, and sporting outside (R^2^ = 0.25). Conclusions: Merely being in nature does not substantially affect well-being. Rather, mindful perception of nature as a space where modern public health practices, such as mindful walking, are particularly effective, is essential. From a public health and urban planning perspective, accessible, quiet, and aesthetically engaging green spaces that attract and fascinate people should be prioritized to foster such restorative experiences.

## 1. Introduction

In recent years, nature, in particular, has attracted medical attention as a health-relevant resource [1]. Quiet moments in nature can have positive effects, especially for stressed people [2] and for people with reduced mental wellbeing [3]. Relatedness to nature is even discussed as a fundamental psychological need [4]. Indeed, the need to immerse oneself in the beauty of nature and to find a place of silence and peace (which is usually in nature) to gain states of inner peace is one of the strongest spiritual needs of chronically ill, elderly, or stressed people [5].

For public health issues, urban greening is of outstanding importance, particularly in areas with tree canopy. Several studies have found positive effects on health and psychological wellbeing, although not all reported such benefits [6]. Ngyen et al. assumed that “different types of facilities may result in different forms of behavior” [6]. Beyond serving as places for social interaction and emotional wellbeing, public green spaces also provide opportunities for recreation and physical activity. Some are suitable for sports and exercise, while others invite slow walks, relaxation, and moments of wondering awe.

### 1.1. Nature-Relatedness, Experience of Nature, and Psychological Health

Despite growing interest in nature’s health benefits, there is limited research on the subjective experience of nature. Most studies have focused instead on cognitively influenced nature-relatedness or short-term effects after intentional visits to nature, such as during ‘forest bathing’ [7].

Nature-relatedness describes positive attitudes towards nature as a habitat [8,9]. Individuals who feel connected to nature have greater psychological wellbeing and can use this resource to improve stress and negative mood [8,9]. Nevertheless, the health-related aspects of nature-relatedness are quite complex and contradictory in their effects [10], as the specific content is important. Dean et al. [10] found that the joy associated with nature-relatedness is associated with wellbeing, whereas self-identification with nature and an intention to protect nature may be associated with an increase in distress and depressive mood.

Similar results as described above can be found for the concrete experience of nature in terms of stress reduction [11,12,13], improved wellbeing, and better psychological mood [14,15,16,17,18,19]. These experiences focus on the direct perception and sensory engagement with the green environment. All the rich details come to one’s awareness and make one pause in amazement, i.e., being touched by the warmth of the sun, light raindrops, a gentle breeze, the rustling of leaves, etc. [20]. Nature is thus understood as a perceptive resource. These moments of wonder and sensory awareness are associated with feelings of inner peace and enjoyment, and may thus enhance wellbeing [20].

### 1.2. Feelings of Wondering Awe in Nature

The availability of green spaces, particularly in urban areas, is often linked to the individual health of stressed people, but is also relevant for public health [21]. However, not only access but also how individuals engage with nature, their inner attitude, and how they ‘resonate’ with it, appears to be crucial. This is of particular importance as a study by Zhang et al. [22] showed that a positive relationship between closeness to nature and psychological wellbeing can only be found if one is sensitized to the beauty of nature. This sensitivity may manifest as wondering awe, which can be triggered by various nature experiences [23].

Wondering awe in this context describes a reaction towards specific situations or emotionally touching experiences that may result in feelings of gratitude [23]. Feelings of awe may be characterized by altered time perception, self-diminishment (in terms of egocentric views), connectedness, perceived vastness, physical sensations, and need for accommodation [24]. Nevertheless, there are also small moments of wonder and fascination [23], not only the rare, vast experiences that change a person’s life with a need for accommodation as suggested by Keltner and Haidt [25]. Nevertheless, the more intense the reflective experiences are, the more they may result in changes in attitudes and behaviors [26,27,28].

Qualitative analyses revealed that the underlying triggers of awe can be categorized as (1) Nature, (2) Persons, (3) Unique Moments, and (4) Aesthetics, Beauty, and Devotion, where nature as a trigger was most often stated. The respective experiences of wondering awe resulted in “feelings of interconnectedness, prosocial behavior, mindful awareness, and contribute to a person’s meaning in life and wellbeing”, and can thus be a health-relevant resource [23].

During the COVID-19 pandemic-related lockdowns, many people rediscovered nature as a ‘space to escape’ from their fears and worries, and enjoyed these times of silence and reflection [3,29,30]. At that time, experiencing nature and enjoying quiet moments mediated the relationship between wondering awe and psychological wellbeing as a dependent variable [3].

Awe represents an accessible and trainable resource. Fascination may be the first step, followed by pausing in wondering awe as the next step. These reactions occur in both religious and non-religious individuals, are typically stronger among older adults, and are most often nature-related [23].

Awe as a resource could be of general relevance for public health, as it was suggested to increase wellbeing [23,31,32], is related to positive emotions and less anxiety [33], gratitude [32], and to personal change [32,34]. The subsequent feelings of gratitude, particularly when it becomes a life orientation, contribute to wellbeing and positive social relationships [35].

### 1.3. Aim of the Study

The aim of this non-interventional cross-sectional study was thus to analyze (1) whether and to what extent both the experience of nature and nature-relatedness are associated with psychological wellbeing, and (2) which role wondering awe and gratitude may play in their interaction.

It is assumed that the experience of nature matters more than merely the availability of green space. It may further make a difference how one faces nature, either just as a place to do some sporting activities or fast cycling, or as a space of mindful encounter while slowly walking and relaxing. Therefore, the influence of these activities will be further analyzed.

## 2. Materials and Methods

### 2.1. Recruitment of Participants

For this anonymous cross-sectional survey, potential participants were informed about the study’s purpose between September 2023 and April 2024 through social networks, student groups, yoga communities, educational courses, and the authors’ professional networks. Participants were invited to disseminate the respective information and link to the online survey in terms of a snowball sampling. The sample is thus a convenience sample that, due to the recruitment method, does not claim to be representative. Most participants were recruited during harvest time (92%), 7% during winter, and 1% during the following springtime.

Participants expressed their consent by clicking the informed consent field on page 2 of the online questionnaire (via LimeSurvey). The study was approved by the Ethical Commission of the University Witten/Herdecke (#S-306/2023).

### 2.2. Sociodemographic Data

At the end of the questionnaire, participants were asked to provide additional information about themselves (gender identity, age, partner status, education level) and where they live (categorized as their ‘living areas’: rural vs. cities or other), access to ‘green spaces’ (parks, meadows, forests), and their accessibility.

In this context, participants were also asked how much attention they pay to nature in their environment (none at all, hardly at all, moderately, a lot, very much). This additional information is used as a descriptive variable.

### 2.3. Standardized Measures

All questionnaire modules were provided in the German language. They are described below.

#### 2.3.1. Experience of Nature

The Experience of Nature Scale (ENS) was used to capture different experiences related to nature [20]. The ENS uses 11 items that can be differentiated into three main factors: (1) Everyday detachment/Relaxation (Cronbach’s α = 0.87), (2) Fascination with nature/Wondering (Cronbach’s α = 0.82), and (3) Sense of responsibility for nature (Cronbach’s α = 0.85). All items were introduced with the phrase “When I am in nature…” (supplemented by the note “e.g., walking, hiking, cycling”). Example statements are, i.e., “… I enjoy being able to escape from all the stresses and strains of everyday life”; “… I notice many ‘small’ details that make me pause in amazement”; “… it motivates me to be mindful of the environment”. The corresponding statements were rated using a 5-point Likert scale (0—strongly disagree; 4—strongly agree). The scales were calculated as mean scores, representing the level of which corresponds to the degree of agreement. The internal consistency of the ENS in this sample is good (Cronbach’s α = 0.89).

#### 2.3.2. Nature Relatedness

To measure nature-relatedness, the 6-item Nature-Relatedness Scale (NR-6) by Nisbet & Zelenski [36] was used. This scale uses four items from the self-scale (#5, #7, #17, #21) of the 21-item extended version [11], none from the perspective-based scale, and two items from the experience-based scale (#4, #9). In consultation with Elizabeth K. Nisbet some phrases were modified (e.g., instead of the formulation “remote wilderness”, “a remote place of untouched nature” was used; instead of “I notice wild animals wherever I am”, “I perceive animals in nature wherever I am” was used). Respondents were asked to rate their agreement or disagreement with the statements using a 5-point Likert scale (0—strongly disagree; 4—strongly agree). A mean score was calculated, the level of which corresponds to the degree of agreement. The scale has good internal consistency in this sample (Cronbach’s α = 0.82).

#### 2.3.3. Awe and Gratitude

Perceptions of wondering awe and subsequent feelings of gratitude were assessed with the 7-item Awe/Gratitude scale (GrAw-7) [32]. It has good psychometric properties (Cronbach’s alpha = 0.82) and uses items such as “I stop and am captivated by the beauty of nature,” “I pause and remain captivated by the moment,” “In certain places I become completely still and reverent,” or “I pause and then think of so many things for which I am truly grateful.” Agreement was assessed on a 4-point scale (0—never; 1—rarely; 2—often; 3—regularly) and then referred to a 100-point scale. The scale’s internal consistency in this sample is good (Cronbach’s α = 0.88).

#### 2.3.4. Gratitude

McCullough’s 6-item Gratitude Questionnaire (GQ-6) addresses dispositional gratitude [37]. Example statements are “I have so much in life to be thankful for” or “As I get older, I find myself more able to appreciate the people, events, and situations that have been part of my life history”. It is scored on a 7-point scale from strong disagreement (1) to strong agreement (7) with two reversely coded items. The internal consistency in this sample is acceptable (Cronbach’s α = 0.71).

#### 2.3.5. Wellbeing

To address general psychological wellbeing in terms of mood, vitality, and interests, we relied on the WHO-Five Wellbeing Index (WHO-5) [38]. Statements such as “I felt happy and in a good mood,” “I felt calm and relaxed,” “I felt energetic and active,” “I woke up feeling fresh and rested,” or “My daily life is full of things that interest me” were referred to the last two weeks. The frequency of these experiences was rated on a 6-point scale from “at no time” (0) to “all of the time” (5). Sum scores < 13 indicate reduced wellbeing in terms of possible psychological stress, depression, or burnout. The scale’s internal consistency in this sample is good (Cronbach’s α = 0.86).

#### 2.3.6. Activities

The frequency of activities such as cycling outside, sporting outside, hiking/strolling in nature, meditation (incl. yoga), and praying was assessed as never (0), at least once a month (1), at least once a week (2), and at least once a day (3).

### 2.4. Statistical Analyses

Descriptive statistics, comparisons of subgroups (ANOVA), as well as first-order correlations (Spearman rho), and stepwise regression analyses were calculated using SPSS 29.0.

Missing descriptive variables were not replaced, while missing single values in the psychological measures were replaced by the method of Expectation–Maximization (EM). This method estimates missing data by alternating between predicting missing values (Expectation step) and updating parameter estimates (Maximization step) [39].

To investigate whether Experience of Nature may mediate the relationship between Awe/Gratitude and Wellbeing, we applied a mediation analysis [35]. In this procedure, both the direct effects of Experience of Nature and of Awe/Gratitude on Wellbeing, and the mediation effect of Awe/Gratitude were evaluated.

Due to the exploratory nature of this study, the significance level was set at *p* < 0.01.

Concerning the classification of the strength of the relationship between variables, r > 0.5 is considered a strong correlation, r between 0.3 and 0.5 a moderate correlation, r between 0.2 and 0.3 a weak correlation, and r < 0.2 a negligible or no correlation. The effect size Eta^2^ describes the magnitude of the difference between groups. For the analysis of variance (ANOVA), Eta^2^ < 0.06 is considered a small correlation, between 0.06 and 0.14 a moderate correlation, and >0.14 a strong effect size.

## 3. Results

### 3.1. Description of Participants

The majority of participants were female (74%), had a high educational level (67%), and were living with a partner (70%) (Table 1). Their mean age was 51.0 ± 13.0 (range 19–82) years. More than half stated that they had access to green areas that were accessible, on average, within 5.7 ± 6.4 (0–60) minutes.

Most paid attention to nature in their surrounding area (78%) and enjoyed being outdoors, even in unpleasant weather (82%). Among them, at least once a day, 27% were cycling outside, 15% were performing sporting activities, 34% were hiking/strolling in nature, 26% were meditating (incl. yoga), and 26% were praying.

As shown in Table 1, participants’ Wellbeing scores were in the higher range (59.6 ± 19.3), while their Awe/Gratitude scores (63.7 ± 18.8) were similar to a reference sample (65.3 ± 19.7) [26].

### 3.2. Intensity of Nature Perceptions

Women had significantly higher scores for Nature-Relatedness, Experience of Nature, and Awe/Gratitude than men, while their Wellbeing was higher only in trend (Table 2). The effect sizes were small, except for the ENS subscale Fascination with nature/Wondering, where it was moderate.

Participants of higher age had significantly higher scores for Nature-Relatedness, Experience of Nature, Awe/Gratitude, and slightly also for Wellbeing than younger ones (Table 2).

With respect to the educational level, no significant differences were observed (Eta^2^ between 0.001 and 0.005). Living with a partner did not significantly affect the nature-related variables (Eta^2^ < 0.001), whereas Wellbeing trended to be higher among participants with a partner (*p* < 0.017; Eta^2^ = 0.012).

In contrast, Nature-Relatedness (*p* < 0.001; Eta^2^ = 0.025), Experience of Nature (*p* = 0.008; Eta^2^ = 0.016), and Awe/Gratitude (*p* = 0.008; Eta^2^ = 0.016) scored marginally higher in participants living in rural areas as compared to those from cities, while their Wellbeing did not significantly differ (Eta^2^ = 0.003).

### 3.3. Correlations Between Nature Perceptions, Awe/Gratitude, Wellbeing, and Activities

Literature so far has focused on the rather cognitive concept of Nature-relatedness, while the experiential aspect (“Experience of Nature”) has so far been less often addressed. As shown in Table 3, although both dimensions may be strongly interrelated, they are nevertheless conceptually different. Further, Nature-Relatedness and Experience of Nature are strongly related to Awe/Gratitude, but only marginally with Wellbeing (Table 3).

The time required to get access to green spaces had no relevant effect on either Nature experience or Wellbeing, but was marginally negatively related to Nature-relatedness (Table 3).

As gratitude can be the outcome of awe experiences, we also addressed the associations with gratitude. While dispositional gratitude was moderately related to Awe/Gratitude and Wellbeing, it was at least weakly correlated to Nature-relatedness, and marginally only to Experience of Nature. This underlines that these dimensions are conceptually different.

Cycling outside had no relevant effect on Nature-relatedness, Experience of Nature, Awe/Gratitude, or Wellbeing, while outside sporting activities were at last weakly related to the ENS subscale Everyday detachment/Relaxation, and further to Wellbeing (Table 3). In contrast, hiking/strolling in nature was moderately related to Nature-relatedness and Awe/Gratitude. Meditation practices were moderately associated with Nature-relatedness, the ENS subscale Fascination with nature/Wondering, and Awe/Gratitude, while praying was best related to Awe/Gratitude and less relevant to nature-associated variables or Wellbeing (Table 3).

### 3.4. Mediation Analyses

**Figure 1:**
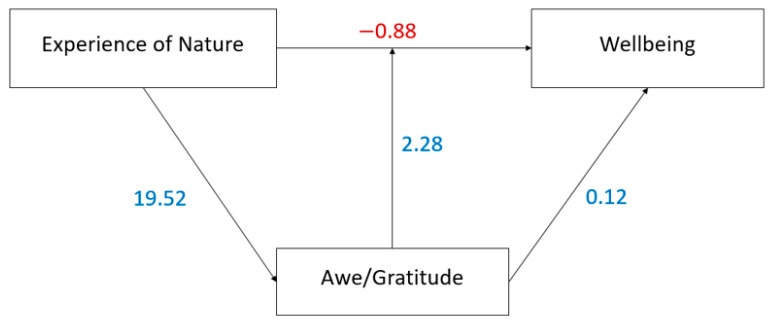
Mediation model with Awe/Gratitude (GrAw-7) as mediator of the link between Experience of Nature (ENS) as the independent variable and Wellbeing (WHO-5) as the dependent variable. Depicted are standardized beta values. Blue numbers indicate positive effects, while the red number indicates a negative effect.

Mediation analyses (Figure 1) revealed that Awe/Gratitude as a mediator exerted a direct positive effect on Wellbeing as the dependent variable (β = 0.12, *p* < 0.001), while experiencing nature as the independent variable showed a direct, but inverse effect on Wellbeing (β = −0.88, *p* = 0.05). This indicates a suppression effect, meaning that the apparent weak or adverse association turns into a positive one once Awe/Gratitude is accounted for, thereby underscoring its indispensable mediating role in translating nature experiences into psychological wellbeing. The overall model explains approximately 29% of the variance in Wellbeing (R^2^ = 0.29). Importantly, Awe/Gratitude served as a significant mediator of the relationship between experiencing nature and Wellbeing. Here, experiencing nature strongly predicted Awe/Gratitude (β = 19.52, *p* < 0.001), and Awe/Gratitude, in turn, predicted Wellbeing (β = 2.28, *p* < 0.001). The total effect of experiencing nature on Wellbeing was β = 1.40 (*p* < 0.001), confirming that the total indirect effect through Awe/Gratitude contributed substantially to this association, thereby confirming its mediating role.

### 3.5. Predictors of Wellbeing

As nature-related variables were not relevantly associated with Wellbeing, while Awe/Gratitude was (Table 3), regression analyses were performed to analyze which of the tested variables could be regarded as predictors of Wellbeing. For that purpose, only significant variables were included, among them the frequency of sporting, hiking/strolling, meditation, and praying.

As shown in Table 4, Wellbeing as the dependent variable was influenced strongest by Awe/Gratitude, gratitude disposition, and sporting outside, far exceeding the explanatory power of other lifestyle factors such as cycling outside, hiking/strolling outside, and even meditation, indicating that emotional-spiritual resources and gratitude play a comparatively greater role in psychological health than these behavioral predictors. However, neither nature-relatedness nor experience of nature alone had significant independent effects. Nevertheless, among these, the subscale Everyday detachment/Relaxation (ENS) was the sole one with a positive influence, while Fascination with nature/Wondering (ENS), Sense of responsibility for nature (ENS), and nature relatedness had negative influences on Wellbeing.

## 4. Discussion

### 4.1. Discussion of Findings

Contrary to our expectations, in this non-interventional study, neither Nature-relatedness nor the Experience of Nature showed a meaningful association with psychological wellbeing. Instead, Awe/Gratitude was moderately related to Wellbeing, and further, strongly to both nature variables. This suggested a mediation effect of Awe/Gratitude, which was confirmed: the ability to perceive moments as unique served as a significant mediator of the relationship between Experience of Nature as an independent and Wellbeing as a dependent variable. This indicates that simply being in a green environment may not necessarily enhance wellbeing when the inner resonance is missing.

The best predictor of wellbeing in this study was Awe/Gratitude, followed by gratitude as a disposition, and further by outside sporting activities. However, none of the nature-associated variables had a significant independent influence on Wellbeing. It became evident that only the ENS subscale “Everyday detachment/Relaxation” had a positive influence on Wellbeing, while all other nature-associated variables had a negative, albeit not significant effect. The same negative relation was observed for Experience of Nature as an independent variable in the mediation analysis, which became positively stronger with Awe/Gratitude as a mediator. It could be assumed that intentionally visiting green nature to achieve states of inner peace and relaxation may indeed have the desired effect. In contrast, a mindful perception of nature might heighten awareness of its fragility, evoking feelings of fear and responsibility for its protection, which in turn may reduce wellbeing. Indeed, Dean et al. [10] suggested that the joy stemming from nature-relatedness contributes to wellbeing, while self-identification with nature and a protective attitude toward it may be related to heightened distress and depressive symptoms.

While the meta-analysis of Capaldi et al. [14] proved that being related to nature may be associated with happiness, the direct connection was usually weak, as observed in this study for wellbeing, too. Rather, it is the way one encounters nature, with what emotional state, and with what intention. This would fit our finding that Awe/Gratitude as a mindful perception of nature mediates the link between nature experience and wellbeing.

Furjes-Crawshaw et al. [40] reported that physical activity in nature had a stronger effect on wellbeing than such activities indoors. Our current findings confirm that the frequency of sporting activities outside had at least a weak association with Wellbeing, and that it is a significant co-predictor of Wellbeing. In contrast, cycling or hiking had only marginal associations with wellbeing. Nevertheless, with respect to cycling outdoors, there were no significant associations with Nature-relatedness, Experience Nature, or Awe/Gratitude, while hiking and strolling had weak to moderate effects but were not related to Wellbeing. Thus, depending on the intention and aim of the activities, the observed effects may differ.

Moreover, experiencing nature and being related to nature could be seen as amplifiers of known public health interventions. As in this study, meditation was moderately related to nature-associated variables, one may assume that this spiritual practice could sensitize one to mindful awareness of the beauty in daily life in general and nature in particular. Indeed, meditation and mindful walking were identified as health-promoting practices in terms of stress reduction and emotion regulation [41,42,43]. Meditation practices may thus sensitize one to the experience of awe and gratitude, which was the best predictor of wellbeing. Mindful walking could be regarded as a low-threshold and inclusive health strategy that reaches different groups of the population, while meditation practices are not accessible to all people and require a strong commitment. Mindful walking [44] or ‘forest bathing’ [45], particularly when it is related to pausing and moments of inner peace and fascination or even wondering awe and gratitude, could be easily accessible resources. Positive effects, particularly on physiological parameters, were regularly reported [18,46].

### 4.2. Implications for Public Health

Public health considers positive health resources as relevant. As such, pausing in wondering awe, which is strongly related to the Experiences of Nature, and subsequent feelings of gratitude, can be regarded as protective factors to promote mental and social health. This resource is not only individually important but also promotes prosocial behavior, connection with others, and compassion [47,48], engagement for others and the environment [49,50]. These behaviors and activities are crucial for healthy societies. While in this non-interventional study, the Experience of Nature alone has no relevant direct effect on wellbeing, it is mediated by Awe/Gratitude. This indicates that the expected positive effects require resonance with nature as a space of encounter. The resulting emotions are key resources for a healthy society.

To integrate these findings into existing public health structures, one may consider different areas: For urban planning, in addition to outdoor places for sporting activities, green spaces for ‘slow experiences’ are also required, particularly for older people. Health departments and community health services may encourage breaks and time-outs for people in green areas to reduce stress and increase their resilience. In schools, mindful nature walks could be integrated into the curriculum to promote concentration and an attitude of gratitude.

### 4.3. Limitations

In contrast to nature-related interventions (such as forest bathing or mindful walking in nature), the participants of this study were not advised to report their experiences after an intentional visit to green spaces. The results may be different if participants consciously visit a special place and develop an emotional connection with it. The findings of such a study are currently in preparation.

In this study, we did not differentiate the types of ‘nature’, and only generally asked for “green spaces (parks, meadows, forests)” and the time to reach them. On average, these green spaces were accessible for most within some minutes. Nevertheless, the free text answers inform us about the places that moved and touched the participants.

Further, we were less successful in activating men and people from a lower educational level, as in this sample, women and people with a higher educational level were predominant. With 74%, women were predominating in this cross-sectional study. However, in a further study where individuals with a preference for nature participated in a guided tour to specific places in nature, similarly, 69% were women (manuscript in preparation). In that second study with a self-selected sample of visitors interested in the “Seelenorte” of the Sauerland region (n = 114), the mean age was 56.8 ± 13.6, and thus slightly higher than in this cross-sectional study with a mean age of 51.0 ± 13.0. In that study, also, the proportion of participants with a university education was high (51%), although less high than in this study. The findings are nevertheless not representative of the German society. Instead, people with an interest in these issues were dominating.

As the data of this study are cross-sectional, no causal interpretations can be drawn.

## 5. Conclusions

The findings of this study indicate that nature is a space that may cause people to pause in fascination and wonder. However, a direct effect of non-interventional experiences of nature or nature-relatedness on psychological wellbeing was not observed; instead, a mediation effect of pausing in wondering awe and gratitude. The related self-transcendent emotions are key factors for resilience and social cohesion—and psychological wellbeing. This makes nature a universally accessible public health resource. Nevertheless, the conditions remain to be further clarified. A current study of our group indicates that in guided visits to unique green places (to focus their awareness), both the experience of nature and feelings of awe/gratitude are triggered, resulting in a direct effect on wellbeing.

## Figures and Tables

**Table 1:** Description of participants (*n* = 491).

	*n* *	%	Mean ± SD
Gender	478	100	
Female	353	74	
Male	120	25	
Other/non-binary	5	1	
Mean age (years)	404		51.0 ± 13.0 (19–82)
Educational level	477	100	
None/primary	8	2	
Secondary classes 8–10	67	14	
Secondary classes 11–13	41	9	
Evening school/Technical college	41	9	
University of Applied Science/University	320	67	
Partner status	476	100	
With partner	332	70	
Without partner	144	30	
Living area	485	100	
Rural	214	44	
Other/cities	271	56	
Access to green spaces	483	100	
Yes	272	56	
No/insecure	211	44	
Access to green spaces (minutes)	478		5.7 ± 6.4 (0–60)
Attention to nature in the surrounding area	482	100	3.1 ± 0.8 (0–4)
never/hardly anything	14	3	
moderate	95	20	
much	211	44	
very much	162	34	
Enjoying being outdoors, even in unpleasant weather (NR item 1)	489	100	3.1 ± 1.0 (0–4)
disagree strongly	6	1	
disagree a little	41	8	
neither agree nor disagree	46	9	
agree a little	193	40	
agree strongly	203	42	
Activities			
cycling outside	478		1.6 ± 1.1 (0–3)
sporting outside	472		1.5 ± 1.0 (0–3)
hiking/strolling	480		2.1 ± 0.7 (0–3)
meditation	480		1.5 ± 1.2 (0–3)
praying	477		1.2 ± 1.3 (0–3)
Psychological Wellbeing (WHO-5)	490		14.9 ± 4.8 (0–25)
Awe/Gratitude (GrAw-7)	491		63.7 ± 18.8 (0–100)

* not all participants provided complete information (the mean percentage of missing differentiating data is 3.5%).

**Table 2:** Intensity of perceptions and experience related to age and gender.

		Nature Relatedness (NR6)	Experience of Nature (ENS)	ENS—Everyday Detachment/Relaxation	ENS—Fascination with Nature/Wondering	ENS—Sense of Responsibility for Nature	Awe/Gratitude (GrAw-7)	Wellbeing (WHO-5)
All participants (*n* = 474)	mean	3.07	3.35	3.29	3.45	3.28	63.82	14.91
	SD	0.72	0.52	0.59	0.60	0.78	18.96	4.86
Age categories								
<31 (*n* = 44)	mean	2.67	3.10	2.99	3.33	2.93	57.03	13.43
	SD	0.79	0.61	0.71	0.68	0.93	18.15	4.78
31–40 (*n* = 63)	mean	2.93	3.23	3.23	3.23	3.22	59.71	14.75
	SD	0.86	0.59	0.66	0.72	0.79	17.83	4.45
41–50 (*n* = 89)	mean	3.00	3.32	3.29	3.39	3.24	61.63	13.87
	SD	0.73	0.55	0.56	0.68	0.77	20.04	5.20
51–60 (*n* = 166)	mean	3.21	3.43	3.36	3.56	3.37	65.51	15.57
	SD	0.62	0.48	0.54	0.53	0.76	18.51	4.70
>60 (*n* = 112)	mean	3.15	3.39	3.31	3.51	3.35	68.03	15.42
	SD	0.67	0.45	0.57	0.49	0.71	18.56	4.87
F value		6.36	4.72	3.73	4.37	3.15	4.28	3.21
*p* value		<0.001	<0.001	0.005	0.002	0.014	0.002	0.013
Eta^2^ value		0.051	0.039	0.031	0.036	0.026	0.035	0.027
Gender *								
women (*n* = 353)	mean	3.16	3.43	3.35	3.56	3.36	65.81	14.73
	SD	0.67	0.48	0.57	0.50	0.74	18.27	4.89
men (*n* = 120)	mean	2.82	3.15	3.15	3.17	3.10	58.22	15.58
	SD	0.76	0.58	0.63	0.74	0.82	19.46	4.63
F value		21.00	27.26	9.89	43.33	10.01	14.97	2.79
*p* value		<0.001	<0.001	0.002	<0.001	0.002	<0.001	0.095
Eta^2^ value		0.043	0.055	0.021	0.084	0.021	0.031	0.006

* as the number of non-binary participants was too small, they were not included in this analysis.

**Table 3:** Correlation analyses.

	Nature Relatedness (NR6)	Experience of Nature (ENS)	ENS—Everyday Detachment/Relaxation	ENS—Fascination with Nature/Wondering	ENS—Sense of Responsibility for Nature	Awe/Gratitude (GrAw-7)	Wellbeing (WHO-5)
Nature-related variables							
Nature Relatedness (NR6)	1.000						
Experience of Nature Scale (ENS)	0.695 **	1.000					
ENS—Everyday detachment/Relaxation	0.576 **	0.888 **	1.000				
ENS—Fascination with nature/Wondering	0.627 **	0.840 **	0.621 **	1.000			
ENS—Sense of responsibility for nature	0.523 **	0.684 **	0.414 **	0.489 **	1.000		
Enjoying being outdoors (NR1)	0.398 **	0.292 **	0.267 **	0.311 **	0.129 **	0.262 **	0.174 **
Access to green spaces (minutes)	−0.157 **	−0.075	−0.033	−0.084	−0.080	−0.085	−0.015
Wellbeing indicators							
Awe/Gratitude (GrAw-7)	0.555 **	0.545 **	0.437 **	0.551 **	0.375 **	1.000	
Wellbeing (WHO5)	0.164 **	0.185 **	0.178 **	0.186 **	0.077	0.416 **	1.000
Dispositional gratitude (GQ-6)	0.227 **	0.160 **	0.087	0.222 **	0.132 **	0.390 **	0.319 **
Activities							
Cycling outside	0.088	0.094	0.049	0.059	0.167 **	0.034	0.111
Sporting outside	0.155 **	0.187 **	0.205 **	0.143 **	0.094	0.141 **	0.230 **
Hiking/strolling outside	0.326 **	0.242 **	0.218 **	0.242 **	0.118	0.301 **	0.168 **
Meditation (incl. yoga)	0.395 **	0.319 **	0.267 **	0.333 **	0.178 **	0.308 **	0.156 **
Praying	0.209 **	0.188 **	0.156 **	0.174 **	0.156 **	0.321 **	0.160 **

** *p* < 0.001 (Spearman rho); moderate (yellow) and strong (orange) associations are highlighted.

**Table 4:** Predictors of Wellbeing (regression analyses).

Dependent Variable: Wellbeing (WHO-5) Inclusion Model: F = 15.5, *p* < 0.001; R^2^ = 0.254	Beta	T	*p*
(constant)		2.246	0.025
Awe/Gratitude (GrAw-7)	**0.355**	**6.238**	**<0.001**
Gratitude disposition (GQ-6)	**0.231**	**5.194**	**<0.001**
Nature relatedness (NR-6)	−0.081	−1.254	0.210
Everyday detachment/Relaxation (ENS)	0.037	0.680	0.497
Fascination with nature/Wondering (ENS)	−0.077	−1.249	0.212
Sense of responsibility for nature (ENS)	−0.040	−0.794	0.428
Sporting outside	**0.168**	**3.942**	**<0.001**
Hiking/strolling outside	0.043	0.964	0.335
Meditation (incl. yoga)	−0.009	−0.201	0.841
Praying	0.036	0.818	0.414

Significant influences were highlighted (bold).

## Data Availability

Due to the data protection and related legal requirements, data can be made available on substantiated request.

## Abbreviations

The following abbreviations are used in this manuscript:

ENS	Experience of nature
WHO-5	WHO-Five Wellbeing Index

## References

[1] Antonelli M., Barbieri G., Donelli D. (2019). Effects of forest bathing (shinrin-yoku) on levels of cortisol as a stress biomarker: A systematic review and meta-analysis. Int. J. Biometeorol..

[2] Pfeifer E., Fiedler H., Wittmann M. (2020). Increased relaxation and present orientation after a period of silence in a natural surrounding. Nordic J. Music. Ther..

[3] Büssing A., Recchia D.R., Baumann K. (2022). Experience of nature and times of silence as a resource to cope with the COVID-19 pandemic is mediating the effects of Awe and Gratitude on psychological Wellbeing—Findings from a continuous cross-sectional survey in Germany. Front. Public Health.

[4] Hurly J., Walker G.J. (2019). Nature in our lives: Examining the human need for nature relatedness as a basic psychological need. J. Leisure Res..

[5] Büssing A. (2021). Spiritual Needs in Research and Practice: The Spiritual Needs Questionnaire as a Global Resource for Health and Social Care.

[6] Nguyen P.-Y., Astell-Burt T., Rahimi-Ardabili H., Feng X. (2021). Green Space Quality and Health: A Systematic Review. Int. J. Environ. Res. Public Health.

[7] Li Q. (2022). Effects of forest environment (Shinrin-yoku/Forest bathing) on health promotion and disease prevention—The Establishment of “Forest Medicine”. Environ. Health Prev. Med..

[8] Nisbet E.K., Zelenski J.M., Murphy S.A. (2009). The Nature Relatedness Scale: Linking individuals’ connection with nature to environmental concern and behaviour. Environ. Behav..

[9] Nisbet E.K., Zelenski J.M., Michalos A.C. (2014). Nature Relatedness and Subjective Wellbeing. Encyclopedia of Quality of Life and Wellbeing Research.

[10] Dean J.H., Shanahan D.F., Bush R., Gaston K.J., Lin B.B., Barber E., Franco L., Fuller R.A. (2018). Is Nature Relatedness Associated with Better Mental and Physical Health?. Int. J. Environ. Res. Public Health.

[11] Fuller R.A., Irvine K.N., Devine-Wright P., Warren P.H., Gaston K.J. (2007). Psychological benefits of greenspace increase with biodiversity. Biol. Lett..

[12] Van Den Berg A.E., Custers M.H. (2011). Gardening promotes neuroendocrine and affective restoration from stress. J. Health Psychol..

[13] Roe J.J., Thompson C.W., Aspinall P.A., Brewer M.J., Duff E.I., Miller D., Mitchell R., Clow A. (2013). Green space and stress: Evidence from cortisol measures in deprived urban communities. Int. J. Environ. Res. Public Health.

[14] Capaldi C.A., Dopko R.L., Zelenski J.M. (2014). The relationship between nature connectedness and happiness: A meta-analysis. Front. Psychol..

[15] Berman M.G., Jonides J., Kaplan S. (2008). The cognitive benefits of interacting with nature. Psychol. Sci..

[16] MacKerron G., Mourato S. (2013). Happiness is greater in natural environments. Glob. Environ. Change.

[17] Joye Y., Bolderdijk J.W. (2015). An exploratory study into the effects of extraordinary nature on emotions, mood, and prosociality. Front. Psychol..

[18] Hansen M.M., Jones R., Tocchini K. (2017). Shinrin-Yoku (Forest Bathing) and Nature Therapy: A State-of-the-Art Review. Int. J. Environ. Res. Public Health.

[19] de Keijzer C., Bauwelinck M., Dadvand P. (2020). Long-term exposure to residential greenspace and healthy aging: A Systematic Review. Curr. Environ. Health Rep..

[20] Büssing A., Recchia D.R., Ortiz M. (2024). Validierung eines Fragebogeninstrumentes zur Erfassung des Erlebens von Natur als subjektive Empfindung. Complement. Med. Res..

[21] Browning M.H.E.M., Rigolon A., McAnirlin O., Yoon H. (2022). Where greenspace matters most: A systematic review of urbanicity, greenspace, and physical health. Landsc. Urban Plan..

[22] Zhang J.W., Howell R.T., Iyer R. (2014). Engagement with natural beauty moderates the positive relation between connectedness with nature and psychological Wellbeing. J. Environ. Psychol..

[23] Büssing A. (2021). Wondering Awe as a perceptive aspect of spirituality and its relation to indicators of Wellbeing: Frequency of perception and underlying triggers. Front. Psychol..

[24] Yaden D.B., Kaufman S.B., Hyde E., Chirico A., Gaggioli A., Zhang J.W., Kelner D. (2019). The development of the awe experience scale (AWE-S): A multifactorial measure for a complex emotion. J. Posit. Psychol..

[25] Keltner D., Haidt J. (2003). Approaching awe, a moral, spiritual, and aesthetic emotion. Cognit. Emot..

[26] Gallagher S., Janz B., Reinerman L., Bockelman P., Trempler J. (2015). Liftoff: Towards an exploration of subjective experience. A Neurophenomenology of Awe and Wonder: Towards a Non-Reductionist Cognitive Science.

[27] Cohen A.B., Gruber J., Keltner D. (2010). Comparing spiritual transformations and experiences of profound beauty. Psychol. Relig. Spiritual..

[28] Silvia P.J., Fayn K., Nusbaum E.C., Beaty R.E. (2015). Openness to experience and awe in response to nature and music: Personality and profound aesthetic experiences. Psychol. Aesthet. Creat. Arts.

[29] Pouso S., Borja Á., Fleming L.E., Gómez-Baggethun E., White M.P., Uyarra M.C. (2021). Contact with blue-green spaces during the COVID-19 pandemic lockdown beneficial for mental health. Sci. Total Environ..

[30] Labib S.M., Browning M.H.E.M., Rigolon A., Helbich M., James P. (2022). Nature’s contributions in coping with a pandemic in the 21st century: A narrative review of evidence during COVID-19. Sci. Total Environ..

[31] Krause N., Hayward R.D. (2015). Assessing whether practical wisdom and awe of god are associated with life satisfaction. Psychol. Relig. Spiritual..

[32] Büssing A., Recchia D.R., Baumann K. (2018). Validation of the Gratitude/Awe Questionnaire and Its Association with Disposition of Gratefulness. Religions.

[33] Rankin K., Andrews S.E., Sweeny K. (2019). Awe-full uncertainty: Easing discomfort during waiting periods. J. Posit. Psychol..

[34] Haidt J., Keltner D., Peterson C., Seligman M.E.P. (2004). Appreciation of beauty and excellence. Character Strengths and Virtues.

[35] Wood A.M., Froh J.J., Geraghty A.W. (2010). Gratitude and Wellbeing: A review and theoretical integration. Clin. Psychol. Rev..

[36] Nisbet E.K., Zelenski J.M. (2013). The NR-6: A new brief measure of nature relatedness. Front. Psychol..

[37] McCullough M.E., Emmons R.A., Tsang J.A. (2002). The grateful disposition: A conceptual and empirical topography. J. Personal. Soc. Psychol..

[38] Bech P., Olsen L.R., Kjoller M., Rasmussen N.K. (2013). Measuring Wellbeing rather than the absence of distress symptoms: A comparison of the SF-36 mental health subscale and the WHO-Five Wellbeing scale. Int. J. Methods Psychiatr. Res..

[39] Molenberghs G., Verbeke G., Molenberghs G., Verbeke G. (2005). Multiple Imputation and the Expectation-Maximization Algorithm. Models for Discrete Longitudinal Data.

[40] Furjes-Crawshaw J., Heke I., Jowett T., Rehrer N.J. (2025). The Physical Activity Environment, Nature-Relatedness and Wellbeing. Int. J. Environ. Res. Public Health.

[41] Goyal M., Singh S., Sibinga E.M., Gould N.F., Rowland-Seymour A., Sharma R., Berger Z., Sleicher D., Maron D.D., Shihab H.M. (2014). Meditation programs for psychological stress and Wellbeing: A systematic review and meta-analysis. JAMA Intern. Med..

[42] Gotink R.A., Hermans K.S., Geschwind N., De Nooij R., De Groot W.T., Speckens A.E. (2016). Mindfulness and mood stimulate each other in an upward spiral: A mindful walking intervention using experience sampling. Mindfulness.

[43] Ma J., Williams J.M., Morris P.G., Chan S.W.Y. (2023). Effectiveness of a mindful nature walking intervention on sleep quality and mood in university students during Covid-19: A randomised control study. Explore.

[44] O’Donovan H. (2015). Mindful Walking: Walk Your Way to Mental and Physical Wellbeing.

[45] Li Q. (2018). Forest Bathing: How Trees Can Help You Find Health and Happiness.

[46] Hanson S., Jones A. (2015). Is there evidence that walking groups have health benefits? A systematic review and meta-analysis. Br. J. Sports Med..

[47] Rudd M., Vohs K.D., Aaker J. (2012). Awe expands people’s perception of time, alters decision making, and enhances Wellbeing. Psychol. Sci..

[48] Piff P.K., Dietze P., Feinberg M., Stancato D.M., Keltner D. (2015). Awe, the small self, and prosocial behavior. J. Pers. Soc. Psychol..

[49] Büssing A., Recchia D.R., Dienberg T. (2018). Attitudes and behaviors related to Franciscan-inspired spirituality and their associations with compassion and altruism in Franciscan brothers and sisters. Religion.

[50] Zhao H., Zhang H., Xu Y., He W., Lu J. (2019). Why are people high in dispositional awe happier? The roles of meaning in life and materialism. Front. Psychol..

